# Advances in the study of tertiary lymphoid structures in the immunotherapy of breast cancer

**DOI:** 10.3389/fonc.2024.1382701

**Published:** 2024-04-02

**Authors:** Xin Li, Han Xu, Ziwei Du, Qiang Cao, Xiaofei Liu

**Affiliations:** ^1^ The First Clinical School of Shandong University of Traditional Chinese Medicine, Jinan, China; ^2^ Innovation Research Institute of Traditional Chinese Medicine, Shandong University of Traditional Chinese Medicine, Jinan, China; ^3^ Department of Earth Sciences, Kunming University of Science and Technology, Kunming, China; ^4^ Department of Breast and Thyroid Surgery, Affiliated Hospital of Shandong University of Traditional Chinese Medicine, Jinan, China

**Keywords:** breast cancer, tertiary lymphoid structures, microorganisms, immunity, machine learning

## Abstract

Breast cancer, as one of the most common malignancies in women, exhibits complex and heterogeneous pathological characteristics across different subtypes. Triple-negative breast cancer (TNBC) and HER2-positive breast cancer are two common and highly invasive subtypes within breast cancer. The stability of the breast microbiota is closely intertwined with the immune environment, and immunotherapy is a common approach for treating breast cancer.Tertiary lymphoid structures (TLSs), recently discovered immune cell aggregates surrounding breast cancer, resemble secondary lymphoid organs (SLOs) and are associated with the prognosis and survival of some breast cancer patients, offering new avenues for immunotherapy. Machine learning, as a form of artificial intelligence, has increasingly been used for detecting biomarkers and constructing tumor prognosis models. This article systematically reviews the latest research progress on TLSs in breast cancer and the application of machine learning in the detection of TLSs and the study of breast cancer prognosis. The insights provided contribute valuable perspectives for further exploring the biological differences among different subtypes of breast cancer and formulating personalized treatment strategies.

## Introduction

Since 2019, breast cancer has surpassed lung cancer as the malignant tumor with the highest global incidence (1), and its incidence is closely related to socioeconomic development, with the highest risk of disease in economically transformed regions and the lowest survival rates in economically underdeveloped regions (2). Underdiagnosis, misdiagnosis and lack of effective treatments are the most important reasons for the huge differences in prevalence and survival rates worldwide (3). The current treatment options for breast cancer include various modalities such as surgery, radiation, immunization, endocrine, targeted,chemotherapy and Chinese medicine. Immunotherapy has made significant breakthroughs in recent years, with immune checkpoint inhibitors in particular boosting survival in many patients. However, this approach has shown significant results in only a subset of patients, with relatively low success rates. Objective remission rates in the vast majority of clinical studies were less than 20%, while median progression-free survival and overall survival were less than 3 and 24 months (4). Therefore, there is a need for in-depth research on new methods and ideas for the treatment of breast cancer. TLSs are a class of ectopic lymphoid organs that form in non-lymphoid tissues and are common in conditions such as tumors, chronic inflammation, and autoimmune diseases. Because TLSs are formed in non-lymphoid tissues, they have a more flexible ability to respond to local abnormal lesions, thus playing an important immunomodulatory role in chronic inflammation and tumor development and contributing to the efficiency of immune response and local therapeutic efficacy. The presence of TLSs has been observed in a variety of malignant tumors such as non-small cell lung cancer, colorectal cancer, breast cancer, melanoma, sarcoma, and renal cell carcinoma (5). Recent findings indicate that researchers are gradually recognizing the positive role of TLSs in treating breast cancer patients. These insights have provided clinicians with new perspectives to reformulate breast cancer treatment protocols from an immune perspective (6). Machine learning belongs to a kind of artificial intelligence, with the arrival of the era of big data, the ability of computers to quickly process complex and huge data is increasingly dominant, and in recent years the increasing popularity of high-throughput histological data and the success of artificial intelligence technology has made machine learning in a variety of fields widely used (7–9). Machine learning is more efficient than humans in predicting and prognosticating treatment for some cancers. This review summarizes the current understanding of TLSs in breast cancer and the potential application of machine learning in the study of TLSs and breast cancer.

## Detecting TLSs through machine learning

The most common techniques for detecting TLSs are multiple immunofluorescence, hematoxylin and eosin (HE) staining, of which multiple immunofluorescence is difficult to generalize in research due to its high cost, small field of view, and high complexity. In contrast, HE staining is easier and remains the clinical standard in histopathology. TLSs have been detected by pathologists on HE slides, but manual detection is time-consuming and laborious, and results vary according to the level of expertise.Li, Z et al. (10) developed a machine-learning-based computational tool for the automatic detection and quantitative assessment of TLSs on routine HE slides. They confirmed its independent prognostic value in an international multicenter cohort of 1924 patients with six common gastrointestinal cancers. An important advantage of this method is the automated enumeration and quantitative characterization of TLSs. This study, the largest to date, confirms the association of TLSs with the survival of patients with gastrointestinal cancers.

## Composition of TLSs

TLSs contain within them T cells, B cells, dendritic cells, and high endothelial venules (HEV) (11). Similar to SLOs, both share the components of B cells, T cells, and dendritic cells. However, compared to SLOs, TLSs have a simpler organization with scattered lymphocyte aggregates and lack the connective tissue membrane of SLOs (12). The non-enveloped structure of TLSs allows the immune cells to fully interact with the surrounding microenvironment, and, unlike SLOs, TLSs do not persist in a specific location, but rather are formed under specific pathologic conditions, independent of secondary lymphoid organ regions. TLSs are formed under specific pathological conditions, independent of secondary lymphoid organ regions, and trigger immune responses under the regulation of clear inflammatory signals. However, the mechanism that triggers the formation of TLSs remains unclear (13).

### T cells, B cells and dendritic cells

The T-cell zone is located in the periphery of TLSs and consists mainly of clusters of T cells and mature dendritic cells; the B-cell zone is located in the central region of TLSs and consists of a large number of B cells and some T cells, follicular dendritic cells, and macrophages. In the T-cell zone, T cells are activated by stimulation with specific antigens, and dendritic cells capture and present antigens so that T cells can recognize and bind to them. Activated T cells proliferate rapidly to form effector T cells, which subsequently migrate to the site of infection to execute an immune attack (14–16). In the B-cell region, B cells are activated and differentiate into plasma cells that produce specific antibodies against antigens. Similarly, dendritic cells capture and present foreign antigens to B cells in this process, initiating antibody production. This coordinated immune response mechanism ensures the body’s immune balance (17–21). Researchers comprehensively analyzed 69 studies covering 19 types of cancers and showed a positive correlation between tumor-infiltrating B cells (including plasma cells) and clinical outcome in half of the cases, while the rest showed either a negative correlation (9.3%) or a neutral effect (40.7%) (22). Helmink and others investigated the density and distribution of B cells and their relationship with TLSs and found that the same properties of memory B cells and plasma cells required for the acquired immune response may contribute to an effective T cell response after neoadjuvant immune checkpoint blockade. Importantly, these B cells may act by altering T cell activation and function and through other mechanisms with key immune components of TLSs (23). Evidence suggests that B cells predict a better prognosis and a higher response rate to immunotherapy only if they form TLSs, and that B cells outside of TLSs may suppress antitumor immunity and promote tumor growth (24). These studies reveal that B cells in the tumor microenvironment not only correlate with clinical outcomes, but may also play a critical role in T cell responses in the context of immunotherapy.

## HEV

HEV is a unique vascular structure in the lymphatic system, which helps the formation of TLSs through its unique morphology and function, and contributes to lymphocyte colonization and immunoregulation in the lymphatic system. Most of the current opinions believe that the formation of TLSs is highly dependent on SLOs and HEV (25–28). Some of the lymphocytes in SLOs spread to tumor tissues through lymphatic vessels or HEV when stimulated by inflammatory factors for a long period of time, thus initiating the formation of TLSs (29). In a retrospective cohort study, Martinet analyzed data containing 146 patients with invasive breast cancer and showed that the density of HEV was positively correlated with patients’ disease-free survival, metastasis-free survival, and overall survival, demonstrating their important role in the formation of TLSs (30). Therefore, tumor HEV may be potential therapeutic targets in cancer diagnosis and treatment. For example, Colbeck and his team effectively killed tumor cells in the Foxp3DTR mouse model by depleting Treg cells to promote the self-expanding circuit of T-cell activation as well as the formation of HEV in the tumor, which in turn induced the formation and maturation of TLSs in the tumor (31).

## The formation process of TLSs

### CXCL13 and IL-7 are involved in the formation of TLSs

**Figure 1:**
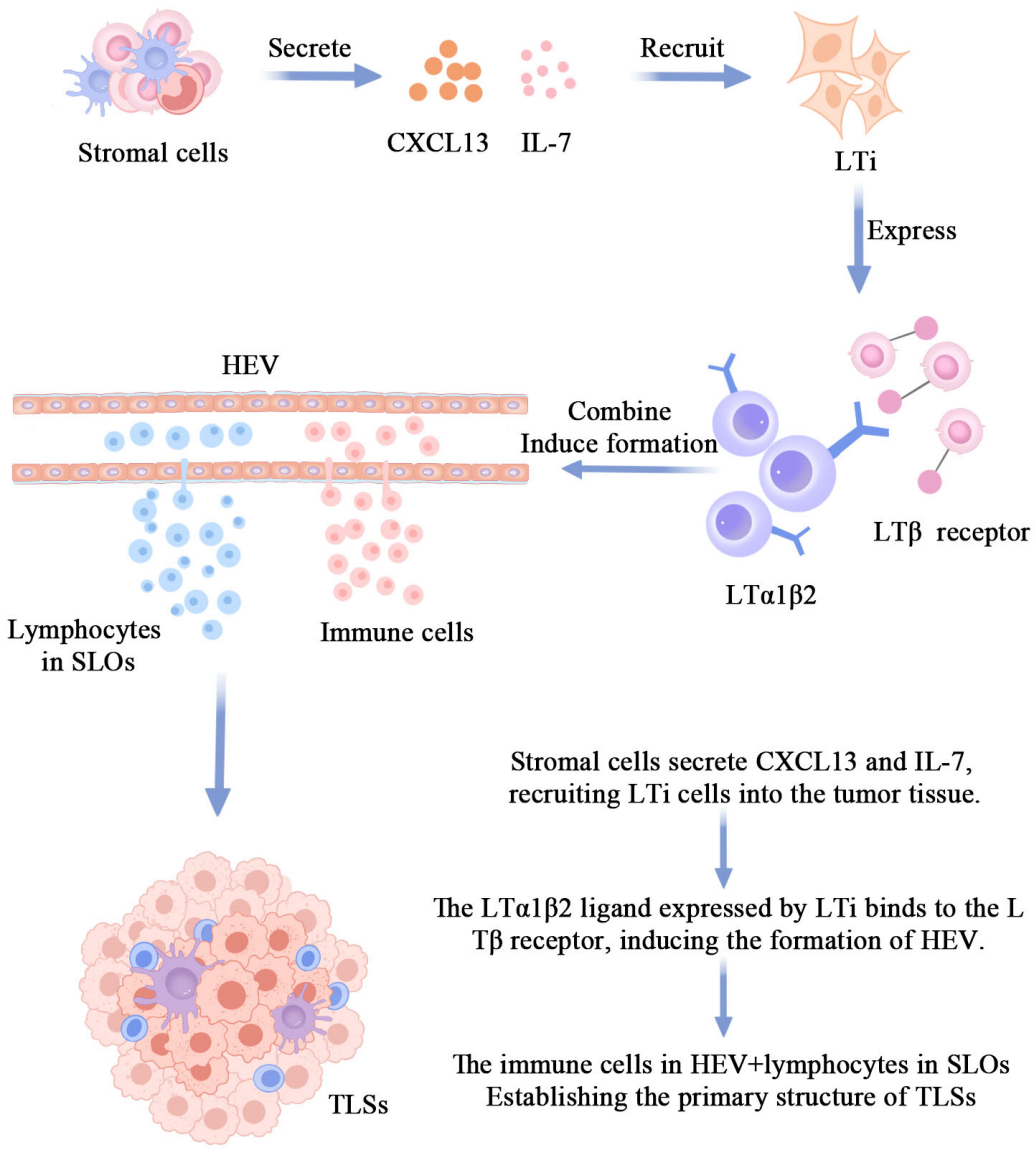
The mechanism of TLS formation.The primary pathway for TLSs formation involves stromal cells secreting CXCL13 and IL-7 to recruit LTi cells into the tumor tissue. The interaction between the LTa1β2 ligand on LTi cell surfaces and the LTβ receptor induces the formation of HEV. Immunocytes within HEV and some lymphocytes in SLO, under prolonged stimulation by inflammatory factors, disseminate into the tumor tissue, giving rise to the initial morphology of TLSs.

The body undergoes a complex series of reactions in response to damaged tissues or pathogens in an inflammatory state. This process involves the release of signaling molecules (e.g., inflammatory cytokines, chemokines, growth factors) from damaged tissues at the site of inflammation, which then attracts surrounding immune cells toward the damaged area (32, 33). Chemokine C-X-C motif ligand 13 (CXCL13) and interleukin (IL)-7 are key chemokines. They are released near the site of inflammation and can direct the migration of lymphoid tissue inducer cells (LTi) (**Figure 1**). CXCL13 recruits B cells to the tumor region to form TLSs, and the investigators revealed that, in the tumor microenvironment, Th-CXCL13 cells clustered in the central B-cell region and formed within the follicular TLSs. co-localization. Notably, they may attract B cells through chemotaxis as a way to promote the formation and maturation of follicular TLSs (34). CXCL13 has been identified as one of the most potent predictors of improved survival in human cancers (35–38), and the production of CXCL13 by T cells infiltrating inflammatory tissues may be a critical step in the initiation of TLSs formation (39). Some studies have confirmed the presence of dense TLSs and tumor-infiltrating lymphocytes (TILs) in clear cell renal cell carcinoma and demonstrated the oncogenic role of CXCL13 expression in clear cell renal cell carcinoma (40), and Hsieh analyzed the disease-free survival of 794 breast cancer patients (Disease-Free Survival, DFS) in 794 breast cancer patients and the clinical correlation between CXCL13 showed that CXCL13 expression was positively correlated with DFS in breast cancer patients, especially in the HER2 group. Researchers went on to investigate 996 breast cancer patients receiving neoadjuvant chemotherapy and showed that CXCL13 expression was highly correlated with complete remission in HER2 patients (41), a finding that could help in the development of new immunotherapies. These studies have confirmed that CXCL13 expression induces the formation of TLSs.IL-7 is a pluripotent cytokine that maintains the homeostasis of the immune system and plays a crucial role in the development, proliferation, and differentiation of T cells as well as in the promotion of B-cell maturation through the activation of the IL-7 receptor (42–46). The expansion of the lymphatic vessels associated with TLSs occurs in two distinct phases. The first stage of expansion is dependent on IL-7. the second stage is responsible for the drainage of leukocytes from the gland and is regulated by lymphotoxin (LT) βR signaling. Maria Iolyeva have demonstrated that autocrine signaling for IL-7 on lymphatic endothelial cells in SLOs regulates lymphatic vessel remodeling and expansion (47). In addition, fibroblast-derived IL-7 may support lymphangiogenesis in a paracrine manner (48), and all of these findings could suggest that IL-7 is a key regulatory molecule for lymphatic vessel expansion in TLSs (49). Indeed, prophylactic blockade of IL-7 affects lymphatic endothelial cell proliferation, which determines the formation of smaller caliber lymphatic vessels. Although this defect is not complete, it could still indicate that IL-7 plays a role in the early stages of lymphangiogenesis associated with TLSs.

### LTi’s involvement in the formation of TLSs

LTi is a special class of lymphocytes that plays an important role in induction during embryonic development and lymphoid organogenesis.LTi expresses many molecules related to lymphoid organogenesis, such as tumor necrosis factor-associated activation-inducing cytokines, nuclear factor kappa B receptor activator (50–54). Besides these proteins, LTi expresses molecules that bind to the LTβR receptor on the surface of stromal cells. LTi also secretes IL-17, which stimulates stromal cells to release a variety of chemokines, including CXCL12, CXCL13, CCL19, and CCL21 (55–58), which in turn attracts more lymphocytes to accumulate in the region where TLSs form. Cupedo and his team found that intradermal injection of nascent LTi induced TLSs (59). Ectopic expression of LTα in pancreatic islets also induces the formation of TLSs, and Picarella found that LTα-deficient mice completely lacked peripheral lymphoid organs (60); in contrast, specific expression of the LTα transgene in the kidney and pancreas triggered severe chronic inflammation with concomitant formation of TLSs that had the ability to promote antigen-specific responses and antibody class switching.In addition, in contrast to overexpression of only LTα, simultaneous overexpression of both LTα and LTβ leads to more pronounced formation of TLSs compared to overexpression of only LTα (61).

## Application of machine learning in breast cancer

Existing research suggests that machine learning is increasingly used for clinical cancer diagnosis, grading, genetic alteration prediction, and disease prognosis, and that it can identify molecular markers for cancer treatment (62–67), predict surgical outcomes (68), and interpret electrocardiograms. Liu, X et al. developed a novel breast cancer recurrence and metastasis risk assessment framework from histopathology images using image features and machine learning techniques (69), The study is expected to reduce the workload of pathologists and improve the chances of survival for breast cancer patients. Sammut, S.-J. analyzed breast tumors from patients with primary invasive cancers who participated in the TransNEO study at Cambridge University Hospitals NHS Foundation Trust between 2013 and 2017, and found that machine-learning models combining clinical, molecular, and numerical pathology data for predicting response to treatment significantly outperformed models based on clinical variables, emphasizing the The importance of data integration for response prediction and can be used to generate similar predictors for other cancers (70). Machine learning can not only diagnose and assess the prognosis of breast cancer from a pathologic perspective, but also help clinicians choose breast cancer treatments from an imaging perspective. Yu, Y. et al. (71) then developed an efficient preoperative magnetic resonance imaging imaging histology for assessing axillary lymph node status in breast cancer using machine learning techniques, which can identify patients with axillary lymph node metastasis in early-stage invasive breast cancer preoperatively. While machine learning has made significant progress in the diagnosis, treatment, and prognosis assessment of breast cancer, it also has limitations. The performance of machine learning algorithms highly depends on the quality and quantity of input data. Therefore, incomplete or biased datasets may affect the accuracy of the model.

## Microbiota and Immunological Research in Breast Cancer

The human microbiota refers to the collection of microorganisms that inhabit the human body. While most microorganisms are beneficial to the human body, an imbalance in the microbial community, where harmful bacteria outnumber beneficial ones, can lead to various diseases, including cancer. In recent years, researchers have uncovered previously unrecognized connections between immune microenvironment dysregulation and breast cancer (72–76). There is limited knowledge about the microbial composition associated with normal breast tissue and breast-related diseases. Human breasts are not sterile; they harbor diverse bacterial communities. Studies have confirmed that, besides the skin, some microbial communities in breast tissue can also translocate from the gastrointestinal tract through the nipple, possibly facilitated by breastfeeding and/or oral contact through sexual activity (77). This breast microbiota stimulates resident immune cells to maintain healthy breast tissue. Additionally, the types of bacteria present and their metabolic activities, such as their ability to degrade carcinogenic substances, may also contribute. Xuan, C. observed that the baseline expression of antimicrobial response genes in tumors is lower than in healthy breast tissue. Microbial DNA is present in the breast, suggesting that bacteria or their components may influence the local immune microenvironment (78). Banerjee et al. examined the specific and common viral, bacterial, fungal, and parasitic features of each breast cancer subtype. They identified distinct patterns in triple-negative and triple-positive breast cancer samples, while ER-positive and HER2-positive samples exhibited similar microbial characteristics. These features, unique or shared among different breast cancer types, offer a new research avenue for gaining further insights into the treatment and prognosis of breast cancer. This provides a novel understanding of the role of the microbiota in breast cancer (79). Research has found that postmenopausal women newly diagnosed with breast cancer exhibit less diversity and compositional differences in their fecal microbiota compared to women without breast cancer. This discovery suggests that the gut microbiota may influence the risk of breast cancer occurrence and could potentially do so through estrogen-independent pathways (80). The stability of the microbiota in the breast is closely intertwined with the immune environment. Clinical practitioners can develop tailored treatment strategies based on the mechanisms of action of the microbiota in the breast in improving the prognosis of breast cancer.

## Mechanisms of TLSs formation involving other factors

Inflammation-related necrosis and macrophage infiltration may be associated with the formation of TLSs. Tumor necrosis is often accompanied by the onset of an inflammatory response in which damaged tissue releases inflammatory factors and cytokines in response to external aggression. In some cases, cell necrosis within the tumor results in the formation of foci of inflammatory necrosis. These necrotic foci become one of the underlying conditions for the formation of TLSs, which attract immune cells (especially macrophages) by releasing a large number of signaling molecules that accumulate in the damaged area (81). The high degree of plasticity of macrophages can adapt to various microenvironmental changes in tissues, and M1-type macrophages, which are usually activated by toll-like receptors, mostly exhibit anti-pro-inflammatory effects in the immune response (82–85). Olson demonstrated that modulation of phagocytosis in macrophages effectively inhibits tumor progression and improves the prognosis of cancer patients (86). Under inflammatory conditions, macrophages activate and release more inflammatory mediators and chemokines, which further direct immune cells to migrate to the region of TLSs, contributing to the structural formation and maintenance of TLSs (87).

## Expression of TLSs in different breast cancer subtypes

TLSs have been shown to have a favorable prognostic function in a variety of malignancies, but have been less well studied in breast cancer. In the available studies, significant differences were found between TLSs-positive and TLSs-negative subgroups within the same molecular subtype of breast cancer, with significant effects on breast cancer recurrence, lymphovascular infiltration and perineural infiltration (88). TLSs in the breast cancer stroma have been associated with activation of tumor angiogenesis, suggesting that this may be a factor favoring breast cancer metastasis, but most of these studies have been performed in animal models and there are no data on human tissues (89).TLSs were detected in 60% of breast cancer tumors and correlated with higher infiltration of TILs by Buisseret. PD-1 and PD-L1 expression were also associated with higher density of TILs and TLSs, in addition TILs density, TLSs and PD-L1 expression were associated with more aggressive tumor characteristics (90), on the basis of this study, researchers found a positive correlation between the expression of immune checkpoint molecules and baseline TILs and TLSs suggesting that assessing these parameters in breast cancer patients may identify immunomodulatory therapies in tumors that are responsive to them (91).

### TLSs and TNBC

With the in-depth study of TLSs in various types of malignant tumors, more and more researchers are finding TLSs in TNBC (92–94). TNBC refers to a subtype of breast cancer that lacks ER, PR, and HER2, which accounts for 15%-20% of breast cancer cases, and more than 50% of patients recur within the first 3-5 years after diagnosis, making it the most malignant subtype of breast cancer (95, 96). Current treatment options are limited to surgery, adjuvant chemotherapy and radiotherapy (97–101). However, these treatments have certain drawbacks: 1. Surgery may result in changes in breast appearance, including reduction or loss of breast size, which may have an impact on the patient’s psychological and emotional well-being. 2. Adjuvant chemotherapy and radiotherapy may cause damage to the immune system. In recent years, immunotherapy has emerged as an innovative treatment option that significantly reduces damage to healthy cells by activating the patient’s own immune system to fight cancer cells (102–105). Available immunotherapies such as PD-1/PD-L1 inhibitors, CTLA-4 inhibitors, and immune-checkpoint combination therapies have dramatically improved the condition of patients with breast cancer and the adverse effects of the treatment.

TLSs have been reported to be present in approximately 60% of breast cancers (106), and most papers aimed at determining the presence of TLSs or their impact on breast cancer prognosis have been reported mainly in the TNBC subtype.Figenschau demonstrated that tumors with higher levels of TILs were associated with the formation of intra-tumoral TLSs, higher tumor grade, and higher degree of inflammation, which led to a poorer prognosis (107). In addition, TLSs were found to correlate with DFS and overall survival in some TNBC patients, providing new ideas for the treatment of TNBC.Schmid found that PD-L0 expression was moderately correlated with TILs in TNBC (r = 45.0-59.8) (108). Researchers showed a positive correlation with TILs in TNBC when using HE-stained slides and CD3/CD20 immunohistochemistry (IHC)-stained slides, by which they found that, compared to TNBC with low plasma cell density, the presence of TLSs in TNBC with high plasma cell density significantly higher numbers (109), in addition they confirmed that TNBC with higher plasma cell density were also associated with higher B-cell density. The investigators collected 108 patients with TNBC treated with neoadjuvant chemotherapy and measured the amount of TILs and TLSs on histopathology using HE-stained slides. HEV densities and subpopulations of TILs by IHC measuring MECA79, CD3, CD8, and CD20, and the amount of TLSs in core needle biopsies from neoadjuvant chemotherapy prior to TNBC using a digital computer analyzer were found to be higher levels of TLSs as represented by MECA79-positive HEV densities, and higher levels of TILs in the HE slides were pathologic complete remission predictors. On the basis of this finding, it was concluded that the approach of increasing HEV may be beneficial for cancer immunotherapy and is of great clinical therapeutic relevance. They also evaluated the percentage of TLSs positivity in all molecular subtypes of breast cancer.Luminal B breast cancer subtype had a high percentage of TLSs positive cases among Luminal subtypes 27.58% followed by Luminal A-BC (24.13%) and Luminal B-HER2 (10.34%) (109).

### TLSs and HER2-positive breast cancer

Studies have shown that the presence of TLSs may be an important good prognostic indicator for patients with HER2-positive breast cancer, regardless of the level of TILs (110). The HER2 receptor belongs to a family of receptors consisting of four cell surface receptors (HER1-4). When expressed at normal levels, HER2 regulates cell growth, differentiation and survival. However, in pathological conditions where HER2 is overexpressed, it leads to aggressive tumor growth. Therefore, the prognosis associated with HER2-positive breast cancer is usually poor and most patients with HER2-positive breast cancer are resistant to targeted hormonal therapy.The most common drugs for treating HER2-positive breast cancer include trastuzumab, lapatinib, pertuzumab, and ado-trastuzumab emtansine. However, there are still questions regarding the optimal sequence, duration, and combination (with or without chemotherapy) of anti-HER2 targeted therapies, both in advanced and adjuvant settings (111–114). Since the presence of TLSs is associated with anti-tumor immune responses and prolonged patient survival, the study of TLSs in HER2-positive breast cancer is of great clinical guidance. It has been shown in the literature that the favorable outcome of many HER2-positive breast cancer patients treated with chemotherapy and/or HER2-targeted therapy is attributed to active anti-tumor immunity, passive immunotherapy (115). Therefore, the presence of TLSs may be an indicator of treatment response in HER2-positive breast cancer patients. It has been found that in HER2-positive patients, the presence of TLSs at the infiltration margins and/or peritumor was associated with better DFS, but not with overall survival (110). Tumor TLSs are surrounded by a specific vascular system, including peripheral lymph node address-positive vessels, which may allow direct migration of peripheral blood lymphocytes into the TLSs. Indeed, primitive T cells and B cells can be found in tumor TLSs, which escape the local immunosuppressive effects of the tumor environment and thus promote more effective antitumor immunity. A significant correlation was found between the extent of TLSs and HER2 IHC score or HER2 gene copy number, as well as a strong correlation between the percentage of precancerous lesions in the ducts and the extent of TLSs (116). It can therefore be hypothesized that increased HER2 protein expression or associated mutations may act as immunogenic factors that attract lymphocytes to the tissue and promote TLSs. Another possible explanation is that the acne necrosis commonly seen in HER2-positive intraductal precancerous lesions may be associated with increased macrophage infiltration, which also plays an important role in the immune response as antigen-presenting cells (117).

## Conclusions

In summary, the in-depth investigation of the relationship between breast cancer and TLSs has brought new ideas and prospects for the clinical diagnosis and treatment of this type of malignant tumor. Machine learning demonstrates high efficiency in the detection of TLSs and the diagnosis and prognosis of breast cancer.Immune checkpoint inhibitors based on TLSs have become an important breakthrough in breast cancer treatment, but not all patients can benefit from them. In the future, we should focus on researching the distribution and activity of TLSs, making effective use of machine learning to identify beneficiary populations, and ensuring more precise selection before treatment. Furthermore, by exploring the molecular signaling pathways influencing the formation and function of TLSs, as well as the microbial characteristics of each subtype of breast cancer, identifying intervention targets, and developing novel treatment strategies, it is possible to formulate personalized treatment plans based on individual patients. This approach has the potential to significantly enhance treatment efficacy and reduce unnecessary medical interventions.

## Author contributions

XinL: Writing – review & editing, Writing – original draft, Validation, Supervision, Methodology, Investigation, Formal analysis, Conceptualization. HX: Writing – review & editing, Visualization, Validation, Supervision, Conceptualization. ZD: Writing – review & editing, Validation, Supervision, Investigation. QC: Writing – review & editing, Supervision, Resources, Investigation, Conceptualization. XiaL: Writing – review & editing, Writing – original draft, Supervision, Resources, Investigation, Funding acquisition.
